# Inclusion of Zinc Oxide Nanoparticles into Virus-Like Peptide Nanocapsules Self-Assembled from Viral β-Annulus Peptide

**DOI:** 10.3390/nano4030778

**Published:** 2014-09-02

**Authors:** Seiya Fujita, Kazunori Matsuura

**Affiliations:** Department of Chemistry and Biotechnology, Graduate School of Engineering, Tottori University, Koyama-minami 4-101, Tottori 680-8552, Japan; E-Mail: saint_arrow1208@yahoo.co.jp

**Keywords:** self-assembly, β-annulus peptide, nanocapsule, ZnO nanoparticle, inclusion

## Abstract

A viral β-annulus peptide connected with a zinc oxide (ZnO)-binding sequence (HCVAHR) at its N-terminal was synthesized, and the inclusion behavior of quantum-sized ZnO nanoparticles into the peptide nanocapsules formed by self-assembly of the peptide in water was investigated. Dynamic light scattering (DLS) measurements showed that ZnO nanoparticles (approximately 10 nm) in the presence of the peptide (0.1 mM) formed assemblies with an average size of 48 ± 24 nm, whereas ZnO nanoparticles in the absence of the peptide formed large aggregates. Transmission electron microscopy (TEM) observations of the ZnO nanoparticles in the presence of the peptide revealed that ZnO nanoparticles were encapsulated into the peptide nanocapsules with a size of approximately 50 nm. Fluorescence spectra of a mixture of the peptide and ZnO nanoparticles suggested that the ZnO surface and the peptide interact. Template synthesis of ZnO nanoparticles with the peptide nanocapsules afforded larger nanoparticles (approximately 40 nm), which are not quantum-sized ZnO.

## 1. Introduction

Nanometer-sized metals, semiconductors and metal oxides have attracted much attention owing to their peculiar physical and chemical properties compared to those of bulk materials [1,2]. Semiconductor nanoparticles (quantum dots), which are a topic of particular interest, are fluorescent nanoparticles that have advantages in brightness and stability and have consequently been applied as bioimaging materials [3,4,5,6]. CdSe nanoparticles, which are representative semiconductor quantum dots, are known to exhibit significant toxicity [7]. In contrast, zinc oxide (ZnO) nanoparticles are known to be less toxic fluorescent quantum dots, whose fluorescent color varies depending on their size and surface defects [8,9,10]. However, the fluorescence of ZnO nanoparticles is unstable in water, because the nanoparticles aggregate. However, the application of silica [11] and polymer [12] coatings to the surface of ZnO nanoparticles improved their stability in water, and the resulting coated ZnO nanoparticles have been used in cell imaging [13].

Ferritin is a natural cage-like protein that performs important roles as a nanocontainer for the storage of ferrous ions [14,15,16]. Because the inside of apo-ferritin is regarded as a nanospace with a diameter of 7 nm, various inorganic nanomaterials, such as metals [17,18,19,20] and semiconductor nanoparticles [21,22,23,24,25,26,27], have been synthesized inside the hollow interior of apo-ferritin. The inorganic nanomaterials are stabilized by being protected by the protein cage. Spherical viruses are another natural protein cage, comprising genome nucleic acids encapsulated in an outer protein shell known as a capsid [28]. Since the viral capsids are an icosahedral protein assembly with discrete nanospace, they have attracted extensive attention as a novel nanocarrier and nanoreactor [29,30,31,32]. To date, the encapsulation of guest nanomaterials, such as dyes [33,34], drugs [35], proteins [36,37], polymers [38], gold nanoparticles [39,40,41], iron oxide nanoparticles [42] and quantum dots [43,44], instead of genome nucleic acids into spherical viral capsids has been reported.

Recently, chemical strategies to rationally design artificial peptide and protein assemblies have been developed [45,46]. We have observed that the synthetic 24-mer β-annulus peptide fragment (INHVGGTGGAIMAPVAVTRQLVGS), which participates in the formation of the dodecahedral internal skeleton, spontaneously self-assembles into virus-like nanocapsules in water [47,48]. We have demonstrated that the interior of virus-like peptide nanocapsules might be cationic at neutral pH and have encapsulated anionic dyes and DNA in the nanocapsules [49]. The pH dependence of the ξ-potentials of the nanocapsules suggests that the C-terminal is directed toward the surface, whereas the N-terminal is directed toward the interior of the peptide nanocapsules. Therefore, the virus-like peptide nanocapsule can be decorated with various functional groups through modification of the peptide’s terminals.

**Figure 1:**
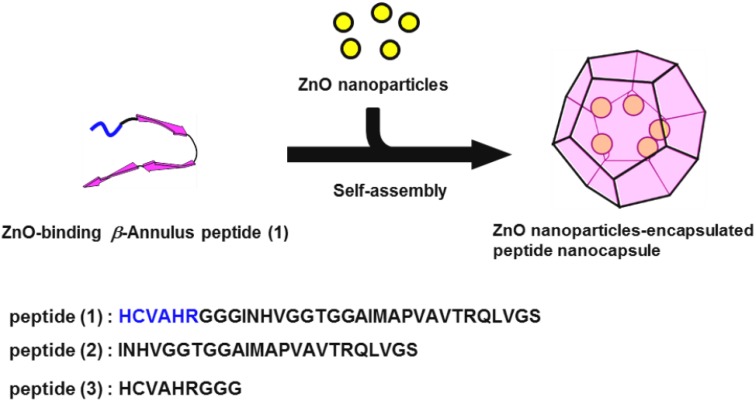
Schematic illustration of the inclusion of ZnO nanoparticles in nanocapsules self-assembled from ZnO-binding β-annulus peptide.

Herein, we designed a novel β-annulus peptide connected via a ZnO-binding sequence at the *N*-terminal, which is directed toward the interior of the peptide nanocapsules. To date, several ZnO-binding peptides have been developed by the screening of peptide libraries [50,51,52,53]. We employed the HCVAHR sequence, developed by Okochi *et al.* [54], as the ZnO-binding peptide, because this short peptide exhibits the highest affinity toward ZnO among the reported ZnO-binding peptides [55]. In this paper, we report the behavior related to the inclusion of ZnO nanoparticles into the virus-like nanocapsules self-assembled from 33-mer ZnO-binding β-annulus peptide **1** (Figure 1).

## 2. Results and Discussion

### 2.1. Synthesis and Self-Assembly of ZnO-Binding β-Annulus Peptide

The 33-mer ZnO-binding β-annulus peptide **1** (HCVAHRGGGINHVGGTGGAIMAPVAVTRQLVGS), whose β-annulus and HCVAHR sequence are linked with a GGG spacer, 24-mer β-annulus peptide **2** and 9-mer ZnO-binding peptide **3** (HCVAHRGGG) were synthesized via the Fmoc-protected solid-phase method using (1-cyano-2-ethoxy-2-oxoethylidenaminooxy)dimethylamino-morpholino-carbenium hexafluorophosphate (COMU) as a condensation reagent; the resulting peptide were purified by reverse-phase HPLC (high performance liquid chromatography) and subsequently confirmed by MALDI-TOF-MS (matrix assisted laser desorption ionization time-of flight mass spectrometry).

**Figure 2:**
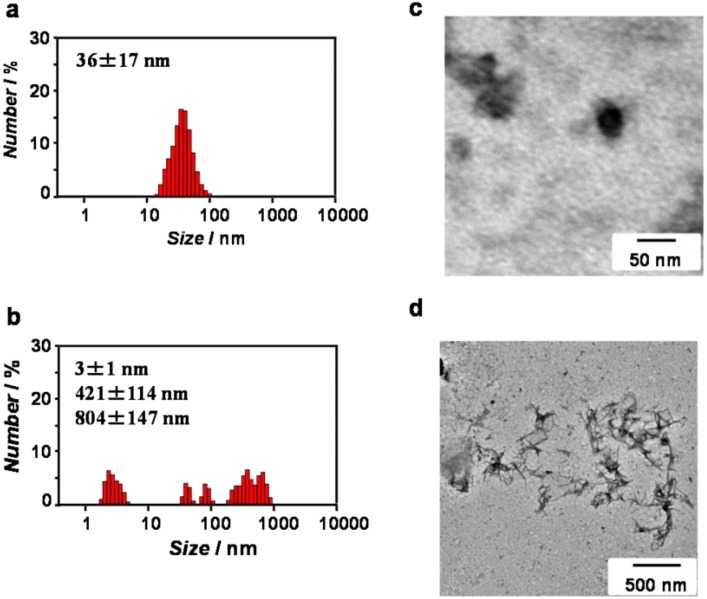
Size distribution obtained from DLS (**a**,**b**) and TEM images (**c**,**d**) for aqueous solutions of peptide **1** at 1.0 mM (**a**,**c**) and 0.1 mM (**b**,**d**) in 10 mM Tris-HCl buffer (pH 7.4) at 25 °C. The TEM samples were stained with sodium phosphotungstate.

ZnO-binding β-annulus peptide **1** was dissolved in 10 mM Tris-HCl buffer (pH 7.4) without heating. The self-assembling behavior of peptide **1** in the buffer was investigated by dynamic light scattering (DLS) measurements and transmission electron microscopy (TEM) observations. The DLS of 1.0 mM peptide **1** in the buffer indicated the formation of assemblies with a size of 36 ± 17 nm (Figure 2a), which is comparable to the size (37 ± 10 nm) of peptide nanocapsules self-assembled from unmodified β-annulus peptide **2** at 1.0 mM [47]. The TEM image stained with sodium phosphotungstate also showed the existence of 30–50 nm spherical assemblies (Figure 2c). In contrast, at 0.1 mM peptide **1**, the DLS revealed a multi-dispersed size distribution (Figure 2b), and the TEM image indicated the existence of irregular fibrous assemblies (Figure 2d). These results indicate that peptide **1** at 1.0 mM self-assembled into the peptide nanocapsules, whereas the nanocapsules were unstable at 0.1 mM. Because β-annulus peptide **2** could form stable nanocapsules at a concentration greater than 25 µM [47], the additional HCVAHRGGG sequence clearly made the nanocapsules unstable.

### 2.2. Preparation and UV-Vis Spectra of ZnO Nanoparticles

**Figure 3:**
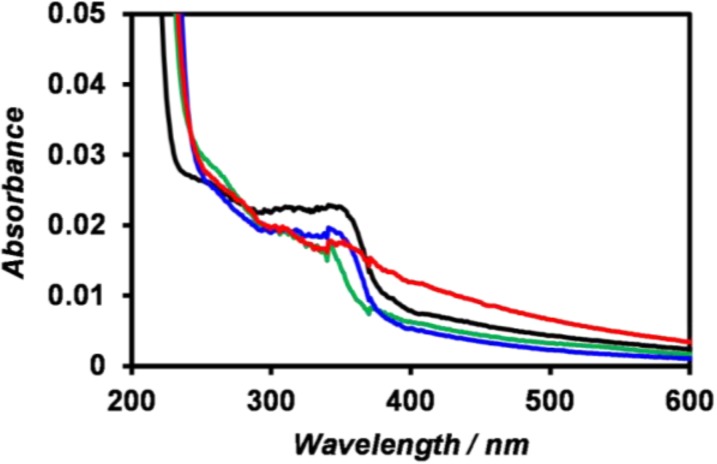
UV-Vis spectra of (black) ZnO nanoparticles, (blue) a mixture of ZnO nanoparticles and peptide **1**, (red) a mixture of ZnO nanoparticles and peptide **2**, and (green) a mixture of ZnO nanoparticles and peptide **3** at [ZnO] = 0.1 mM and [peptide] = 0.1 mM in 10 mM Tris-HCl buffer (pH 7.4) at 25 °C.

ZnO nanoparticles were prepared from Zn(OH)_2_ according to a literature method reported by Uekawa *et al.* [56]. The UV-Vis spectrum of the synthesized ZnO nanoparticles in 10 mM Tris-HCl buffer showed a cliff-shaped spectrum with an absorption peak at 340 nm (Figure 3, black), indicating the formation of ZnO nanoparticles of a quantum size. Meulenkamp reported an experimental Equation (1) of size dependence of the optical band-gap of ZnO nanoparticles, where *d* is the diameter of ZnO nanoparticles and λ_1/2_ is the wavelength at which the absorption is 50% of that at the excitonic peak [57]:

1240λ_1/2_ = 3.556 + 799.9/*d*^2^ − 22.64/*d* (1)

According to the spectrum, λ_1/2_ was 363 nm; thus, the diameter of the ZnO nanoparticles was calculated to be 5.6 nm. However, a gentle-sloping absorption occurred in the wavelength regions longer than 390 nm; thus, the band edge was obscured, indicating coexistence of larger ZnO nanoparticles. The addition of peptides **1**–**3** into the dispersion of ZnO nanoparticles minimally affected the nanoparticles’ UV-Vis spectra (Figure 3).

### 2.3. Inclusion of ZnO Nanoparticles into Peptide Nanocapsules

**Figure 4:**
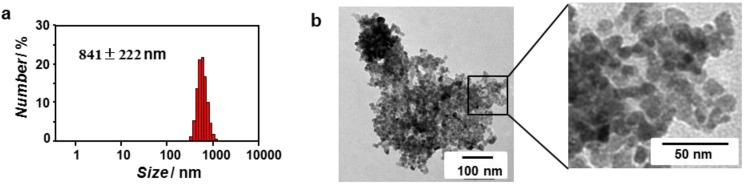
Size distribution obtained from (**a**) DLS and (**b**) unstained TEM image of ZnO nanoparticles ([ZnO] = 0.1 mM) in 10 mM Tris-HCl buffer (pH 7.4) at 25 °C.

The DLS results for the synthesized ZnO nanoparticles ([ZnO] = 0.1 mM) in 10 mM Tris-HCl buffer showed the formation of aggregates of 841 ± 222 nm (Figure 4a). The TEM also showed the formation of large aggregates consisting of ZnO nanoparticles with diameter of 9–15 nm (Figure 4b). These results suggest that the synthesized ZnO nanoparticles were unstable against aggregation in the buffer. The ZnO nanoparticles observed by TEM were larger than the size estimated on the basis of the UV-Vis spectrum, which might indicate that the indistinct band edge results in the underestimation of the nanoparticles’ size.

**Figure 5:**
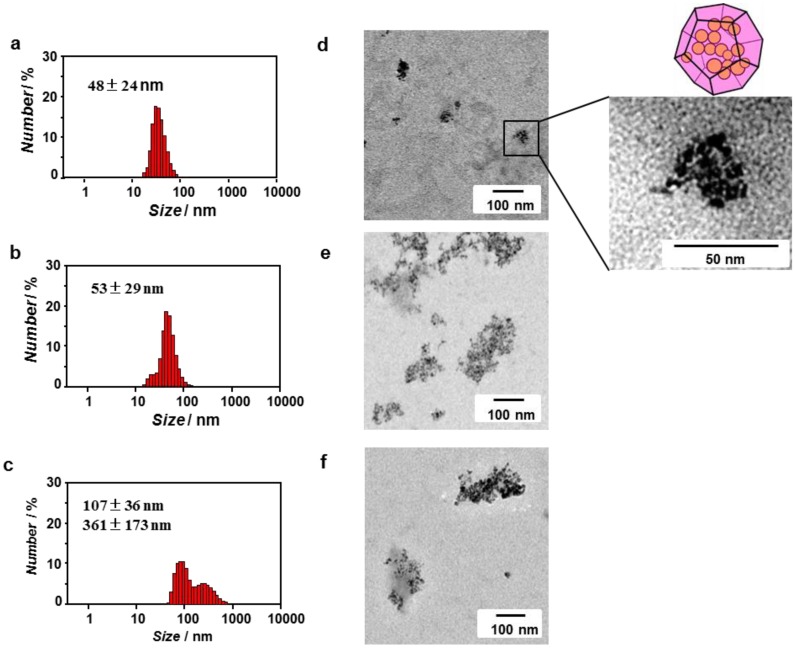
Size distribution obtained from DLS (**a**–**c**) and unstained TEM images (**d**–**f**) of ZnO nanoparticles in the presence of peptides ([ZnO] = [peptide] = 0.1 mM). (**a**,**d**) Mixture of ZnO nanoparticles and peptide **1**; (**b**,**e**) mixture of nanoparticles and peptide **2**; and (**c**,**f**) mixture of ZnO nanoparticles and peptide **3** in 10 mM Tris-HCl buffer (pH 7.4) at 25 °C.

The inclusion behavior of ZnO nanoparticles into peptide nanocapsules formed by self-assembly of the ZnO-binding β-annulus peptide **1** in water was investigated by DLS and TEM. An aqueous dispersion of ZnO nanoparticles in 10 mM Tris-HCl buffer (pH 7.4) was added to the dried peptide **1** (the final concentration: [ZnO] = [peptide] = 0.1 mM), and the mixture was subsequently incubated for 10 min at room temperature. Figure 5a,d shows the size distribution obtained from DLS and TEM images, respectively, of a mixture of ZnO nanoparticles and ZnO-binding β-annulus peptide **1**. Assemblies consisting of several ZnO nanoparticles (individual size: approximately 10 nm) with an average diameter of 48 ± 24 nm were observed without the presence of large aggregates in Figure 4. The diameter is comparable to that of peptide nanocapsules self-assembled from β-annulus peptide **2** [47]. These results indicate that several ZnO nanoparticles with a size of approximately 10 nm are included into the peptide nanocapsules self-assembled from peptide **1**. Because peptide **1** did not form nanocapsules at 0.1 mM, as shown in Figure 2b, the peptide nanocapsules were apparently stabilized by the presence of the ZnO nanoparticles. It is probable that the ZnO-encapsulated peptide nanocapsules formed by the following mechanism: (1) the ZnO-binding sequence HCVAHR of peptide **1** was adsorbed onto ZnO nanoparticles; (2) several ZnO nanoparticles were aggregated; and (3) simultaneously the β-annulus sequence of peptide **1** formed nanocapsule on aggregates of ZnO nanoparticles. In contrast, a mixture of ZnO nanoparticles and β-annulus peptide **2**, which lacks the ZnO-binding sequence, formed larger and irregular aggregates (Figure 5e), although the average diameter obtained from DLS was 53 ± 29 nm (Figure 5b). In addition, a mixture of ZnO nanoparticles and ZnO-binding peptide **3** also formed larger and irregular aggregates (Figure 5f). These results indicate that the formation of 50-nm assemblies consisting of several ZnO nanoparticles is characteristic for ZnO-binding β-annulus peptide **1**. The ZnO nanoparticles likely interact with the ZnO-binding sequence in the peptide nanocapsules self-assembled from peptide **1**. The ZnO nanoparticles encapsulated in nanocapsules self-assembled from peptide **1** remained dispersed for at least five hours, whereas the ZnO nanoparticles in the presence of peptide **2** or **3** aggregated within 10 min.

**Figure 6:**
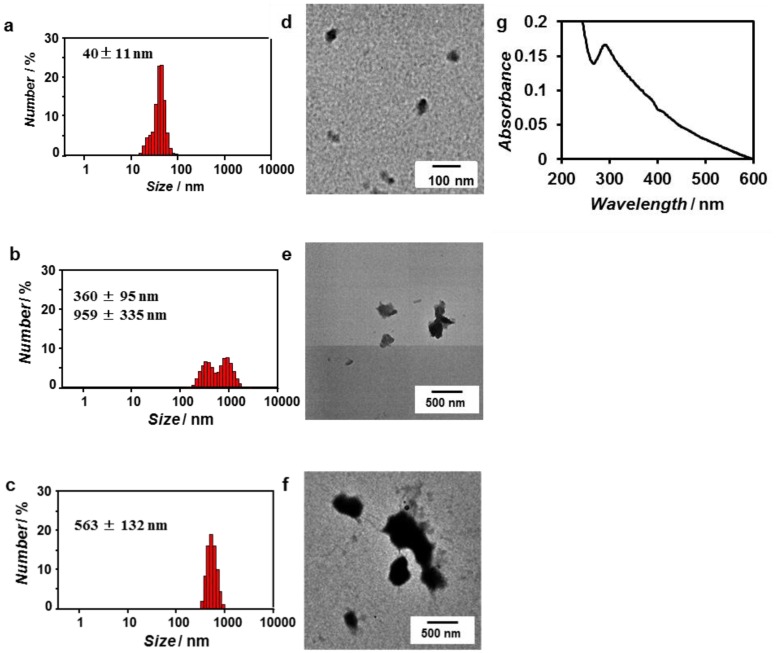
Size distribution obtained from DLS (**a**–**c**), unstained TEM image (**d**–**f**), and UV-Vis spectrum (**g**) of ZnO nanoparticles synthesized in the presence of peptide **1** (**a**,**d**,**g**), peptide **2** (**b**,**e**), and peptide **3** (**c**,**f**) in water.

As shown in Figure 2a, peptide **1** forms nanocapsules with a size of 36 ± 17 nm at 1.0 mM. Therefore, we examined the templated synthesis of ZnO nanoparticles using ZnO-binding β-annulus peptide **1** at 1.0 mM. When Zn(OH)_2_ was dehydrated in the presence of 1.0 mM peptide **1**, ZnO nanoparticles with a size of approximately 40 nm were observed by DLS and TEM (Figure 6a,d), which indicates that peptide **1** acted as a template in ZnO formation. On the other hand, syntheses of ZnO nanoparticles in the presence of peptides **2** and **3** afforded irregular particles with a size of 200–1000 nm. However, the UV-Vis spectrum of the ZnO nanoparticles synthesized in the presence of peptide **1** was characteristic of bulk ZnO (Figure 6g).

### 2.4. Fluorescence Spectra of ZnO Nanoparticles Included in Peptide Nanocapsule

**Figure 7:**
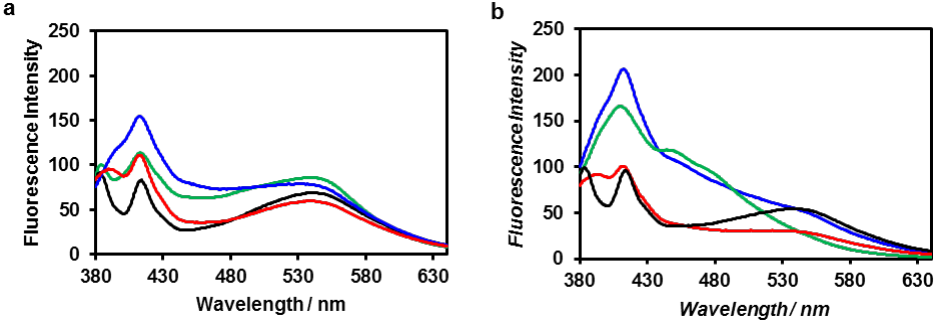
Fluorescence spectra of (black) ZnO nanoparticles, (blue) a mixture of ZnO nanoparticles and peptide **1**, (red) a mixture of ZnO nanoparticles and peptide **2** and (green) a mixture of ZnO nanoparticles and peptide **3** at [ZnO] = 0.1 mM and [peptide] = 0.1 mM in 10 mM Tris-HCl buffer (pH 7.4) at 25 °C; (**a**) immediately after preparation and (**b**) after 24 h. Excitation wavelength: 340 nm.

The fluorescence spectrum of ZnO nanoparticles excited at 340 nm in Tris-HCl buffer showed three emission peaks (Figure 7, black): near-UV emission at 380 nm, which originates from the near-band-edge transition [58]; blue-violet emission at 415 nm, which originates from zinc interstitial defects [58]; and green emission at 540 nm, which originates from the recombination of electrons trapped at singly-ionized oxygen-vacancy defects [59]. The fluorescence spectrum of ZnO nanoparticles in the presence of ZnO-binding β-annulus peptide **1** exhibited an enhanced emission peak at 410 nm, and the peak further increased in intensity after 24 h. The addition of ZnO-binding peptide **3** to the aqueous dispersion of ZnO nanoparticles also resulted in an enhanced emission peak at 410 nm, although the intensity was lower than that in the case of peptide **1**. Such enhancement of blue-violet emission has been also reported by Chang *et al.* [60], who attributed the enhancement to the electronic passivation effect caused by polyaniline modification of the ZnO surface. Therefore, the enhanced intensity of the emission peak at 410 nm shown in Figure 7 is reasonably ascribed to the electronic passivation effect caused by interaction between the ZnO-binding peptide and the ZnO surface. In contrast, the fluorescence spectrum of ZnO nanoparticles in the presence of β-annulus peptide **2** exhibited an only slightly enhanced emission peak at 410 nm, which indicates less interaction between the peptide and ZnO surface. These results suggest that ZnO nanoparticles were included in the virus-like nanocapsules via interaction between ZnO-binding sequence HCVAHR and ZnO nanoparticles.

## 3. Experimental Section

### 3.1. General

Reagents were obtained from a commercial source and used without further purification. Reversed-phase HPLC was performed at ambient temperature on a Shimadzu LC-6AD liquid chromatograph equipped with a UV-Vis detector (220 nm, Shimadzu SPD-20A, Sgimadzu Co., Kyoto, Japan) and GL Science Inertsil WP300 C18 (4.6 mm × 250 mm and 20 mm × 250 mm) columns. MALDI-TOF mass spectra were obtained on an Autoflex II (Bruker Daltonics, Billerica, MA, USA) spectrometer under the linear/positive mode with α-cyano-4-hydroxy cinnamic acid (α-CHCA) and dithranol as the matrix. UV-Vis spectra were recorded at 25 °C using a JASCO V-630 spectrophotometer (Jasco Co., Tokyo, Japan). Fluorescence spectra were measured with excitation at 340 nm at 25 °C using a JASCO FP-8200 spectrophotometer.

### 3.2. Syntheses of Peptides

ZnO-binding β-annulus peptide (**1**): The peptide H-His(Trt)-Cys(Trt)-Val-Ala-His(Trt)-Arg(Pbf)-Gly-Gly-Gly-Ile-Asn(Trt)-His-(Trt)-Val-Gly-Gly-Thr(tBu)-Gly-Gly-Ala-Ile-Met-Ala-Pro-Val-Ala-Val-Thr(tBu)-Arg(pbf)-Gln(Trt)-Leu-Val-Gly-Ser(tBu)-Alko-PEG resin was synthesized on Fmoc-Ser(tBu)-Alko-PEG resin (Watanabe Chemical Ind., Ltd., Hiroshima, Japan, 0.21 mmol/g) using Fmoc-based coupling reactions (4 equivalent Fmoc amino acids). Solutions of (1-cyano-2-ethoxy-2-oxoethylidenaminooxy)dimethylamino-morpholino-carbenium hexafluorophosphate (COMU) and diisopropylamine in *N*-methylpyrrolidone (NMP) were used as coupling reagents. A solution of 20% piperidine in *N*,*N*-dimethylformamide (DMF) was used for Fmoc deprotection. The progress of the coupling reaction and Fmoc deprotection was confirmed using TNBS (2,4,6-trinitrobenzene sulfonic acid) and the Chloranil Test Kit (Tokyo Chemical Industry Co., Ltd., Tokyo, Japan). The peptidyl resin was washed with NMP. The peptide was deprotected and cleaved from the resin by treatment with a mixture of trifluoroacetic acid (TFA)/thioanisole/water/1,2-ethanedithiol/triisopropylsilane = 8.15/0.5/0.5/0.25/0.1 at room temperature for 3 h. The reaction mixture was filtered to remove the resin, and the filtrate was concentrated under vacuum. The peptide was precipitated by adding methyl *tert*-butyl ether (MTBE) to the residue, and the supernatant was decanted. After the washing with MTBE was repeated three times, the precipitated peptide was dried under vacuum. The crude product was purified by reverse-phase HPLC (RP-HPLC, Inertsil WP300 C18, GL Science Inc., Tokyo, Japan), eluting with a linear gradient of CH_3_CN/water containing 0.1% TFA (23.5/76.5 to 25.5/74.5 over 100 min). The fraction containing the desired peptide was lyophilized to give 6.2 mg of a flocculent solid (6.2% yield). MALDI-TOF-MS (matrix: α-CHCA): *m*/*z* = 3181 [M]^+^.

β-Annulus peptide (**2**): **2** was synthesized according to the previously reported procedure [47]. MALDI-TOF-MS (matrix: α-CHCA): *m*/*z* = 2305 [M + H]^+^.

ZnO-binding peptide (**3**): **3** was synthesized using a procedure similar to that described for **1**. The isolated yield was 14.3%. MALDI-TOF-MS (matrix: dithranol): *m*/*z* = 916 [M + Na]^+^.

### 3.3. Syntheses of ZnO Nanoparticle

ZnO nanoparticles were synthesized according to a literature method [55]. A solution of 0.1 M aqueous NH_3_ (0.75 mL) was added to a 0.1 M aqueous solution of Zn(NO_3_)_2_·6H_2_O (0.75 mL). The mixture was stirred for 1 min and then incubated at room temperature for 5 min. The obtained Zn(OH)_2_ precipitate was separated by centrifugation at 10,000 rpm for 1 min. After the obtained precipitate was washed with water two times, the coexisting ammonium ions and nitrate ions in the precipitate were removed. The Zn(OH)_2_ precipitate was dispersed in 0.05 M solution of Zn(NO_3_)_2_·6H_2_O in ethylene glycol (0.75 mL), and the dispersion was subsequently heated at 35 °C for 1 h. After the heating process, ZnO particles were separated by the addition of 0.1 M aqueous NH_3_ (0.75 mL) to the sol. The obtained precipitate was then separated by centrifugation at 10,000 rpm for 1 min, washed with water two times and dried at 75 °C for 12 h. The isolated yield was 2.8 mg (44%).

### 3.4. Inclusion of ZnO Nanoparticles into Peptide Nanocapsules

An aqueous solution of ZnO-binding β-annulus peptide **1** (1 mM, 20 µL) was dried *in vacuo*. An aqueous dispersion of ZnO nanoparticles ([ZnO] = 0.1 mM, 0.2 mL) in 10 mM Tris-HCl buffer (pH 7.4) was added to the dried peptide **1**, and the mixture was incubated for 10 min at room temperature.

### 3.5. Synthesis of ZnO Nanoparticles in the Presence of Peptide **1**

The Zn(OH)_2_ precipitate was prepared using the same procedure described in Section 3.3. The precipitate was separated by centrifugation at 10,000 rpm for 1 min and washed with water two times. The Zn(OH)_2_ precipitate was dispersed in a 0.05 M aqueous solution of Zn(NO_3_)_2_·6H_2_O (7.5 mL). An aliquot (20 µL) of the dispersion was added to dried peptide **1** (2 nmol), and the dispersion ([peptide] = 1.0 mM) was subsequently heated at 35 °C for 1 h. After the heating process, ZnO particles were separated by adding 0.1 M aqueous NH_3_ (0.75 mL) to the sol. The obtained precipitate was then separated by centrifugation at 10,000 rpm for 1 min, washed with water two times and dried *in vacuo*.

### 3.6. Dynamic Light Scattering (DLS) Measurements

DLS was measured with a Zetasizer NanoZS (MALVERN Instruments Ltd., Worcestershire, UK) instrument at 25 °C using an incident He–Ne laser (633 nm). The correlation time of scattered light intensity *G*(τ) was measured several times, and their averaged data were fitted to Equation (2):

(2) $$G(\text{τ})=B+A\exp(-2q^{2}D\text{τ})$$

where *B* is the baseline, *A* is the amplitude, *q* is the scattering vector, τ is the delay time and *D* is the diffusion coefficient. The hydrodynamic radius (*R_H_*) of the scattering particles was calculated by the Stokes–Einstein equation (Equation (3)):

(2) $$R_{H}=\frac{{\text{κ}}_{B}T}{6\text{πη}D}$$

where η is solvent viscosity, *k_B_* is Boltzmann’s constant and *T* denotes the absolute temperature.

### 3.7. Transmission Electron Microscopy (TEM)

An aliquot (5 µL) of each sample solution was applied to a carbon-coated grid (C-SMART Hydrophilic TEM grid, ALLANCE Biosystems, Osaka, Japan), left for 60 s and then removed. The grid was subsequently dried *in vacuo*. In the case of peptide samples, a drop of 2 wt% aqueous sodium phosphotungstate was placed on each of the grids and dried *in vacuo*. The sample-loaded carbon-coated grids were subjected to TEM observation (JEOL JEM 1400 Plus, JEOL Ltd., Tokyo, Japan) using an acceleration voltage of 80 kV. The ZnO nanoparticles were observed without the use of a stain.

## 4. Conclusions

We have demonstrated that a β-annulus peptide having a ZnO-binding sequence self-assembled into the virus-like peptide nanocapsules and included several ZnO nanoparticles inside. ZnO nanoparticles included in the nanocapsules were relatively stable in water, even though ZnO nanoparticles typically tend to aggregates under such conditions. ZnO nanoparticles formed larger aggregates in the presence of β-annulus peptide or ZnO-binding peptide. The fluorescence spectra of ZnO nanoparticles in the presence of ZnO-binding β-annulus peptide revealed that ZnO nanoparticles interact with the peptides. We envisage that the artificial virus-like nanocapsules can include various inorganic materials through proper modification of their interior. We will extend the present molecular design to various peptide-inorganic fusion materials in future work.
